# Xanthogranulomatous Pyelonephritis: A Case Report and Literature Review

**DOI:** 10.1002/ccr3.70774

**Published:** 2025-08-08

**Authors:** Orgeness Jasper Mbwambo, Nelton Rodrick Thobias, Alex Mremi, Jasper Saidi Mbwambo, Frank Bright, Bartholomeo Nicholaus Ngowi

**Affiliations:** ^1^ Department of Urology, Faculty of Medicine Kilimanjaro Christian Medical University College Moshi Tanzania; ^2^ Department of Urology Kilimanjaro Christian Medical Centre Moshi Tanzania; ^3^ Department of Pathology, Faculty of Medicine Kilimanjaro Christian Medical University College Moshi Tanzania; ^4^ Department of Pathology Kilimanjaro Christian Medical Centre Moshi Tanzania

**Keywords:** chronic pyelonephritis, lipid‐laden macrophages, necrotizing inflammation, urinary tract infection, xanthogranulomatous pyelonephritis

## Abstract

Xanthogranulomatous pyelonephritis (XGP) is a rare form of chronic pyelonephritis, often associated with renal calculus and infection *Escherichia coli* and *Proteus mirabilis* are the most frequent organisms isolated in individuals with the disease. Its incidence is 1.4 cases per 100,000 population each year. We present a case of a 69‐year‐old woman who presented with abdominal pain, icterus, fever, non‐projectile vomiting, anemia, and recurrent urinary tract infections. A diagnosis of nephrolithiasis was made and the patient was started on empirical antibiotics. An abdominal pelvic CT scan was performed that showed features suggestive of XGP; a nephrectomy was performed, and a tissue biopsy was taken for histopathology and the results confirmed a diagnosis of XGP. XGP diagnosis is extremely challenging due to its ability to mimic other renal pathologies, and a high degree of suspicion is required. Abdominal pelvic ultrasound is helpful in preoperative diagnosis and a biopsy postoperatively is required to confirm XGP diagnosis. Medical management of XGP with antibiotics and nephrectomy is the best treatment option.

**Summary**

- A high degree of suspicion is required to diagnose xanthogranulomatous pyelonephritis (XGP). Abdominal pelvic ultrasound is helpful in preoperative diagnosis and a biopsy postoperatively to confirm XGP diagnosis.
- Management of XGP with antibiotics and nephrectomy is the best treatment option and they have shown to be associated with the best outcome.

## 1 Introduction

Pyelonephritis is an inflammation of the renal parenchyma primarily due to bacterial infections [1]. Chronic pyelonephritis can result in an unusual form of a disease known as xanthogranulomatous pyelonephritis (XGP) [1]. Patients with this disease usually present with a chronic urinary tract obstruction due to an underlying renal calculus and urinary tract infections [1]. The most frequent organisms extracted from XGP patients are *Escherichia coli* and *Proteus mirabilis* , with different levels of antimicrobial resistance being presented [2].

XGP is marked by extensive damage to renal parenchyma, which is then substituted by a focal collection of xanthomatous mass of lipid‐laden macrophages, also known as foam cells [3]; however, the exact etiology of XGP is still unknown. The disease is very rare; its incidence in the general population is 1.4 cases per 100,000 population each year [4]. The highest incidence is at 50–70 years of age, with a female predominance. The most common clinical manifestations of XGP include anemia, excessive weight loss, abdominal or frank pain, palpable flank mass, and fever [5].

There is usually a challenge in establishing a correct diagnosis of XGP; this is because it mimics other renal pathologies [3]. XGP has acquired the nickname of the “great imitator,” due to the matching of the radiological characteristics of other pathologies including renal cell carcinoma (RCC), malakoplakia, tuberculosis (TB), and urothelial tumors (TCCs) [2].

Management of XGP involves a combination of both medical and surgical approaches. A retrospective study was conducted at St. Vincent's hospital on the outcomes of XGP patients who underwent nephrectomy in their institution for over 12 years period [2]. Bacteriuria was found in most of the patients (91%), with *E. coli* being the predominant organism. Sixty percent of positive urine samples had antibiotic resistance. All patients underwent open nephrectomy, except for one. It was concluded that open nephrectomy is often required in complicated cases of XGP and is associated with high rates of postoperative complications. However, meticulous contemplation of antibiotics and surgical interventions is important to ensure the effective outcome of the patients [2]. Here we share a rare case of a female with XGP.

## 2 Case Presentation (Examination)

A 69‐year‐old female presented to our facility with right‐sided abdominal pain for 3 weeks (3/52); the condition started gradually and progressively worsened with time. She reported that the pain was sharp and pricking, more on the RUQ, constant, and non‐radiating. Associated with nausea, and vomiting which was non‐projectile and consisted of recently consumed food materials, she also had on‐and‐off low‐grade fever, yellowish discoloration of the eyes, and deep yellow‐colored urine. There was no history of diarrhea, heartburn, or painful micturition reported; however, she had a history of repeated UTI and decreased urine frequency reported. The condition had no aggravating factors.

On physical examination, she appeared ill‐looking, jaundiced, pale, and febrile. Her vitals were blood pressure 100/58 mmHg, pulse rate 104 bpm, and oxygen saturation of 94% on room air. On examining the abdomen, she had an obese abdomen with an extended subumbilical midline incision scar, tenderness on the right iliac fossa on deep palpation, hepatomegaly of 7 cm below the right costal margin, no splenomegaly, a tympanic percussion note, and bowel sounds heard and were normal. No remarkable findings were noted upon examination of other systems.

A provisional diagnosis of obstructive jaundice secondary to choledocholithiasis was made with a differential diagnosis of right obstructive ureteric stone and anemia due to infection. The patient was started on empirical antibiotics with broad‐spectrum third‐generation cephalosporin (ceftriaxone) and IV fluids and she was then admitted.

Laboratory investigations were carried out and were as follows: ESR 140 mm/h; alanine aminotransferase (ALT) 65.00 U/L, aspartate transaminase (AST) 41.00 U/L; alkaline phosphatase (ALP) 220 U/L; peripheral blood smear (PBS) showed lysed red blood cells; full blood picture (FBP) showed Hb of 4.5 g/dL; leukocytosis of 28.32 × 10^9^/L with predominance of neutrophils at 87%; conjugated bilirubin 56.79 μmol/L (0.64 mg/dL) (Table 1). Blood culture and sensitivity was performed, which revealed coagulase‐negative staphylococci (CoNS)—*Staphylococcus hemolyticus*—sensitive to vancomycin.

**TABLE 1:** Laboratory tests findings.

SN	Parameter	Test results (units)	Reference range
01	Hemoglobin (Hb)	7.7 (g/dL)	11.5–16.5
02	Serum creatinine (S. Cr)	63 (micromole/L)	44–88
03	Potassium (K)	5.57 (mmol/L)	3.5–5.1
04	Total bilirubin	22.2 (micromole/L)	0.0–21
05	Direct bilirubin	11.00 (micromole/L)	0–3.4
06	Gamma glutamyl transferase (GGT)	285.00 (U/L)	5–36
07	Aspartate transaminase (AST)	30.00 (U/L)	2–32
08	Alanine aminotransferase (ALT)	13.00 (U/L)	2–33
09	Prothrombin time (PT)	1.04 (s)	9.4–12.0
10	Estimated glomerular filtration rate (eGFR)	87 (mL/min)	> 90

The antibiotic was then changed to culture specific, in which vancomycin iv 1 g bid was initiated since the patient still had some episodes of spikes of fever despite being on previous antibiotics. Fever subsided after 1 day of vancomycin, which she was kept on a 7‐day dose; she was also kept on antipain medications, and during the admission after control of infection, she received 3 units of blood transfusion after 3 days. Control labs investigations: Hb—7.7 g/dL, creatinine—63 μmol/L, eGFR—87 mL/min, blood urea nitrogen (BUN)—5.57 mmol/L, sodium (Na)—124; potassium (K)—5.57 mmol/L, total billrubin—22.2 μmol/L, direct bilirubin—11.00 μmol/L, GGT—285.00 U/L, AST—30.00 U/L, ALT—13.00 U/L, prothrombin time (PT)—1.04 (Table 1).

Imaging studies were performed including an ultrasound of the abdomen and pelvis, which showed the left kidney with normal size and echo textures measuring 9.9 cm × 6.7 cm while the right kidney was enlarged in size with multiple complex cysts. So an impression of multiple complex cysts of the right kidney was made with a recommendation of further study with abdominal pelvic CT.

**FIGURE 1:**
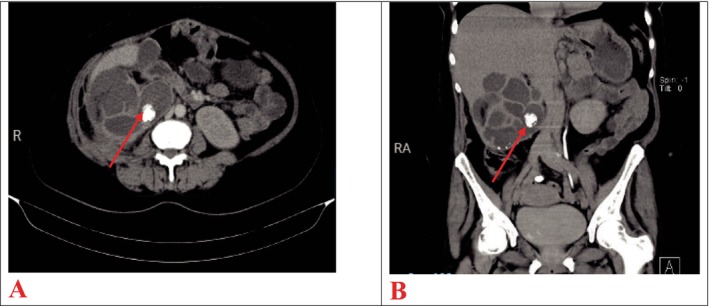
An axial (A) and coronal (B) CT images showing an enlarged, lobulated right kidney with low attenuating cystic spaces and hyperdense lesions (staghorn calculus) shown by the red arrow.

Contrast‐enhanced CT scan of the abdomen revealed an enlarged right kidney with loss of normal outline, appearing lobulated with low attenuating cystic spaces, and multiple hyperdense lesions seen within the kidney (staghorn calculi) the largest measuring 1.8 cm × 2.4 cm in size (Figure 1). Another hyperdense focus was seen in the distal right ureteral outline measuring 1.3 cm × 1.2 cm in size, suggestive of ureteral calculi. A loculated collection in the right posterior perinephric space measuring 5.1 cm × 3.1 cm × 6.4 cm was also noted. An impression of XGP, right ureteral calculi, and approximately 52.9 mL of loculated collection in the right posterior perinephric space was made.

After a confirmed diagnosis of a right complex renal cyst with XGP as well as renal calculi, and after receiving 3 more units of blood, the patient was stable with an Hb of 12 g/dL, with other parameters of the FBC being at normal range, and no episodes of fever spikes. The patient was then planned for a right nephrectomy, which was performed by an experienced urologist and assisted by a urology resident under general anesthesia, with the patient placed in left decubitus position on the operating table. A right subcostal flank incision was made, and the right flank region was opened in layers, encountering edematous muscles. An access to the right retroperitoneal space was gained through blunt dissection after opening a small area at the tip of the right twelfth rib, encountering peri‐nephric fat adhesions, fibrosis, and an inferior vena cava that was adherent to the hilum. The hilum was positioned more superior than usual. Posterolateral mobilization of the lower pole of the right kidney was done to get a plane of dissection, and then carefully a blunt and sharp dissection to further mobilize the kidney was made, encountering adhesions that posed a difficulty in reaching the hilum on the medial aspect. Eventually, the hilum was reached, and the right renal artery was ligated using Vicryl 2‐0 suture, transected, and another suture was transfixed distal to the first ligation. The same was done to the right renal vein. Then, the proximal ureter was also ligated and transected, freeing the grossly disfigured right kidney, which was then placed on a kidney dish (Figure 1). Hemostasis was then achieved, and the abdomen was washed with warm saline. Then, a tube drain was put in place, the flank region was closed in layers using Vicryl 1 suture, and finally, the primary skin incision was closed using nylon 3‐0 suture in a vertical mattress suturing technique.

**FIGURE 2:**
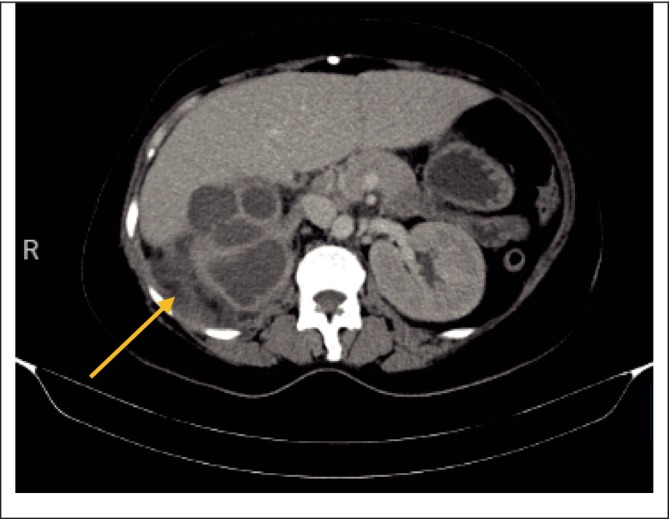
A loculated collection in the right posterior perinephric space shown by the arrow.

**FIGURE 3:**
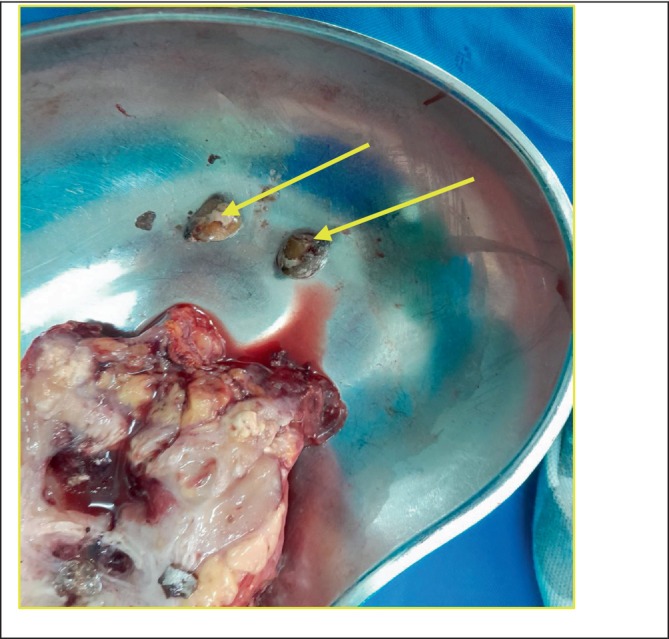
Stones (shown by arrows) that were For Review Only removed from the kidney after cutting it longitudinally in two halves.

**FIGURE 4:**
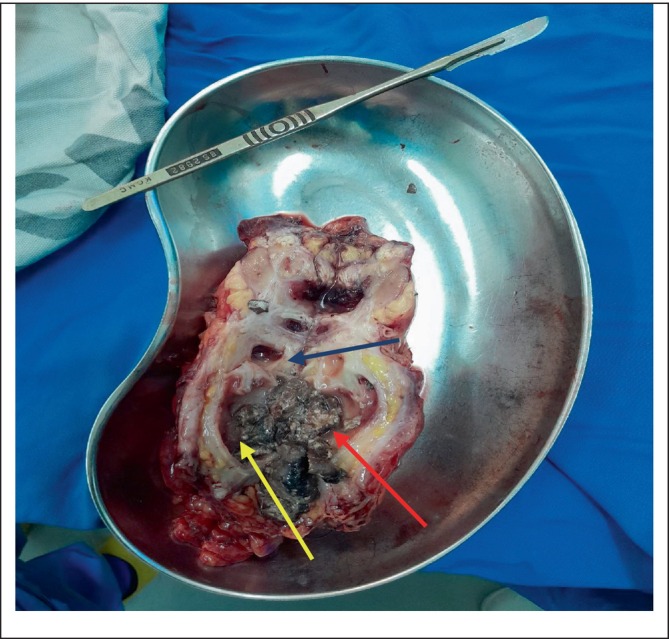
Half of the longitudinally cut right kidney showing some whitish‐yellow areas (red arrow), a pus pocket (blue arrow), and an area of necrotic tissue (yellow arrow).

The removed kidney was cut longitudinally into two halves. A small stone was identified and set aside (Figures 2 and 3). Also present were whitish‐yellow areas, pus pockets, and an area of necrotic tissue, with no identifiable renal tissue (Figures 4).

**FIGURE 5:**
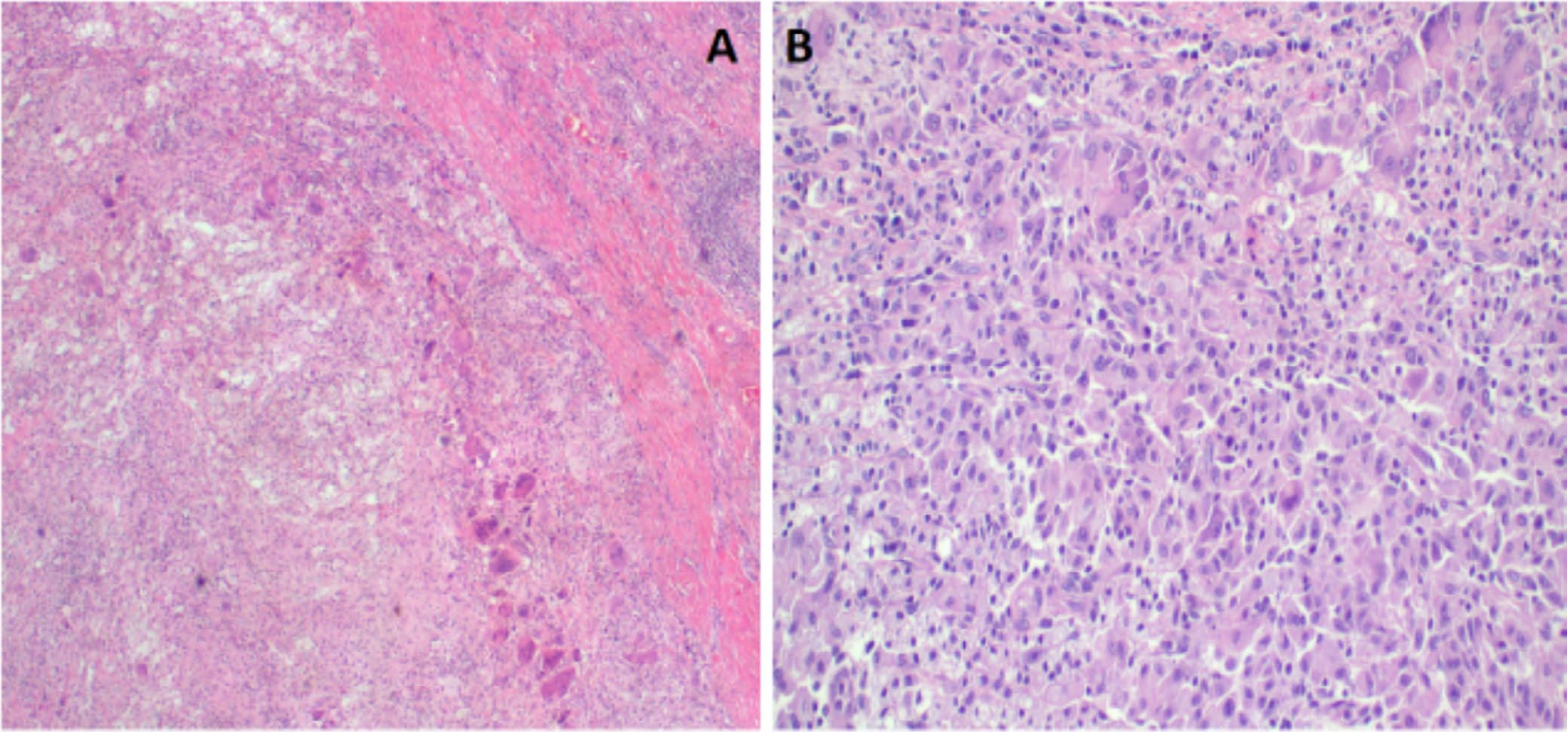
Histopathology of nephrectomy specimen demonstrating zonal distribution of inflammation that includes neutrophils, lymphocytes, plasma cells, xanthoma cells, foreign body giant cells, and fibrous tissue consistent with XGP, H&E staining at 40× original magnification (A); a photomicrograph of XGP highlighting granulation tissues surrounded by lipid laden foamy macrophages (xanthoma cells) and multinucleated giant cells, H&E staining at 100× original magnification (B).

Histopathological evaluation of the specimen was done, whereby microscopically there was a diffuse proliferation of foamy histiocytes replacing renal parenchyma, occasional multi‐nucleated giant cells, and suppurative or necrotizing inflammation features were visualized (Figure 5), it was concluded that the morphology was consistent with XGP and so a diagnosis was made.

The patient stayed for 9 days in the ward. On Days 1 and 2, the drainage was 100 mL of serosanguinous fluid and the wound dressings were dry. Wound inspection was done on Day 3, and the wound was clean and dry on Day 4; however, the patient developed some minimal serosanguinous discharge from the wound but no fever spikes. The wound was managed locally with povidone‐iodine painting and dressing with sterile gauze. Also, from Day 3 through Day 5, drainage had reduced and was at 50 mL per day, while on Day 6, it reduced to only 35 mL of serosanguinous fluid; hence, the drain was removed. Sutures were removed on Day 7, and the patient was discharged home on Day 9 in a good condition with stable vitals.

## 3 Differential Diagnosis

There is usually a challenge in establishing a correct diagnosis of XGP; this is because it mimics other renal pathologies [3]. XGP has acquired the nickname of the “great imitator,” due to the matching of the radiological characteristics of other pathologies including RCC, malakoplakia, TB, and TCCs [2]. Other differential diagnoses of XGP include renal abscesses, and renal and urotherial neoplasms [6].

## 4 Conclusion and Results (Outcome and Follow‐Up)

It is a challenge to diagnose XGP preoperatively because a high degree of suspicion is required. Abdominal pelvic ultrasound is helpful in preoperative diagnosis, and a biopsy postoperatively is required to confirm XGP diagnosis. Medical management of XGP with antibiotics and nephrectomy is the best treatment option, and they have shown to be associated with the best outcome. In our case, the patient underwent right nephrectomy, and the patient stayed for 9 days in the ward. On Days 1 and 2, the drainage was 100 ml of serosanguinous fluid and the wound dressings were dry. Wound inspection was done on Day 3, and the wound was clean and dry on Day 4; however, the patient developed some minimal serosanguinous discharge from the wound but no fever spikes. The wound was managed locally with povidone‐iodine painting and dressing with sterile gauze. Also, from Day 3 through Day 5, drainage had reduced and was at 50 ml per day, while on Day 6, it reduced to only 35 ml of serosanguinous fluid; hence, the drain was removed. Sutures were removed on Day 7, and the patient was discharged home on Day 9 in good condition with stable vitals.

## 5 Discussion

XGP is a chronic granulomatous inflammatory disorder of the renal parenchyma associated with infections and renal calculus. In 1961, Schlagenhaufer was the first man to describe it [7]. The disorder is rare and a different kind of chronic pyelonephritis that frequently appears in the background of the urinary tract obstruction by renal stones and refractory infections of the urinary tract [8]. Its incidence in the general population is 1.4 cases per 100,000 population each year [4]. XGP has the highest incidence in middle‐aged women; however, it can occur at any age [8]. Usually, the disorder presents and affects one kidney (unilateral), but sometimes rarely, it can affect both kidneys (bilateral). Bilateral disease is associated with poor prognosis and is probably due to bilateral loss of renal function [8].

XGP can present with abdominal or frank pain, anemia, palpable frank mass, fever, and weight loss. However, in most cases, XGP has no pathognomic presenting symptoms.

In our case, there was a history of right upper quadrant abdominal pain, icterus, fever, non‐projectile vomiting, anemia, and recurrent urinary tract infections. Choledocolithiasis and cholelithiasis can also occur with similar presentations. It is easy to misdiagnose, and hence the preoperative diagnosis of XGP is challenging.

Imaging helped with the diagnosis of XGP before the operation was performed. XGP can be classified into three categories depending on the extent of the inflammatory condition: focal (only the cortex is involved), segmental (a segment of the kidney is involved), and diffuse (the whole kidney is involved) [9]. According to imaging by CT scan, XGP can be categorized into two forms: focal and diffuse, with the diffuse form being the most frequent one [8].

Diagnosis of XGP can be challenging; there can be a suspicion of the disease preoperatively through the use of imaging techniques, but it cannot be confirmed. CT scan still stands as the first imaging option for the diagnosis of XGP. A “bear paw” sign, which is seen on the cross‐sectional appearance of the kidney tissues on a CT scan, indicating dilation of calyces and contracted renal pelvis, is a characteristic of the disease [10]. Sometimes even a biopsy can still miss the diagnosis, as it can overlook the classic presentation of granulomatous tissue loaded with lipid‐filled macrophages [10].

Preoperative diagnosis of XGP may be a daunting task related to the rarity of its presentation. Thus, scrupulous histopathological evaluation is essential for the definitive diagnosis. In our case, the diagnosis was made suspicious preoperatively by ultrasound and CT scan of the abdominal pelvic region and confirmed postoperatively through histopathological examination whereby microscopically there was a diffuse proliferation of foamy histiocytes replacing renal parenchyma, occasional multi‐nucleated giant cells, and suppurative features; hence, it was concluded that the morphology was consistent with XGP. Although *E. coli* and *P. mirabilis* are the most common bacteria found in association with XGP, our patient's blood culture grew coagulase‐negative staphylococci (CoNS)—*S. hemolyticus*.

Although nephrectomy (open or laparoscopic) is the cornerstone of the treatment of XGP, some cases have been reported to be managed medically, particularly in children [3]. In the state of diffuse disease, total nephrectomy is indicated, whereas in focal disease, partial nephrectomy can be done (nephron‐sparing) [3, 11]. In our case, the patient was managed by nephrectomy and antibiotics for the infection.

## Author Contributions

**Orgeness Jasper Mbwambo:** conceptualization, formal analysis, investigation, resources, supervision, visualization, writing – original draft, writing – review and editing. **Nelton Rodrick Thobias:** writing – original draft, writing – review and editing. **Alex Mremi:** methodology, writing – review and editing. **Jasper Saidi Mbwambo:** writing – review and editing. **Frank Bright:** writing – review and editing. **Bartholomeo Nicholaus Ngowi:** writing – review and editing.

## Consent

Written informed consent was obtained from the patient to publish this report in accordance with the journal's patient consent policy.

## Conflicts of Interest

The authors declare no conflicts of interest.

## Data Availability

The authors have nothing to report.
